# Short Femoral Stem Performance in Femoral Hip Fracture: Clinical and Radiological Evaluation and Comparative Study of Patients Older than 65 Years

**DOI:** 10.3390/medicina62010126

**Published:** 2026-01-08

**Authors:** Daniel Godoy-Monzon, Jose Manuel Pascual Espinosa, Patricio Telesca

**Affiliations:** Hospital San Rafael, Diego Arias 2, 11002 Cadiz, Spain; josepascualespinosa@gmail.com (J.M.P.E.); patricio.telesca@gmail.com (P.T.)

**Keywords:** arthroplasty, femoral neck fractures, hip replacement, short femoral stem, standard femoral stem, outcomes

## Abstract

*Background and Objectives*: Short femoral stems are increasingly used in total hip arthroplasty (THA), yet evidence regarding their performance in elderly femoral neck fracture (FNF) patients is limited. In this study, we compared clinical and radiographic outcomes of the use of a short femoral stem (SFS) versus a conventional standard stem (CSS) in cementless THA. *Materials and Methods*: This prospective, single-center case–control study (1:2) included patients ≥ 65 years of age with displaced FNF (Garden 3–4) treated with cementless THA. Follow-up lasted a minimum of 2 years. Clinical evaluations included the Harris Hip Score (HHS), Roles and Maudsley satisfaction score, and thigh pain assessment. Radiographic evaluations assessed cup position, osseointegration (Moore signs), radiolucencies (DeLee–Charnley and Gruen zones), subsidence, leg length discrepancy (LLD), and heterotopic ossification. *Results*: A total of 114 patients were analyzed (38 with SFS versus 76 with CSS). The final follow-up HHS was 87 ± 2.7 (SFS) and 88 ± 2.5 (CSS) (*p* = 0.231), and satisfaction was excellent in nearly all patients in both groups. Thigh pain was rare and resolved by final follow-up in all SFS patients, and no radiographic loosening was observed. Early subsidence (≤3 mm) occurred in two SFSs and three CSSs without progression, while LLD < 1 cm was present in three SFS and eight CSS cases. No implant-related revisions occurred, and complication rates were low and comparable. *Conclusions*: Short femoral stems provided clinical and radiographic outcomes equivalent to those of conventional stems in elderly FNF patients treated with cementless THA. Short stems appear to be a safe and effective option in this population, and further studies with longer follow-up are needed to confirm their durability.

## 1. Introduction

Total hip arthroplasty (THA), introduced by Sir John Charnley in 1959, is considered the surgical procedure of the century due to its significant improvement in patients’ quality of life and function [1]. Currently, over one million THA procedures are performed annually. In recent decades, uncemented implants have become the gold standard, achieving direct implant–bone union with biological fixation and long-lasting performance [2]. However, aseptic loosening remains the most common cause of revision surgery in the long term, affecting implant survival [3]. Conventional proximal tapered stems have been demonstrated to lead to favorable outcomes in femoral neck fractures [4,5]. Nevertheless, concerns persist regarding the use of conventional cementless stems, particularly the occurrence of thigh pain related to diaphyseal fixation, and the risk of unexpected bone loss associated with stem morphology and an increase in periprosthetic fracture risk [5,6].

Short cementless stems combined with minimally invasive approaches have been increasingly used in total hip arthroplasty, preserving bone, sparing soft tissue, and restoring proximal femoral anatomy more accurately [7,8].

Total hip arthroplasty is considered a better treatment option in displaced femoral neck fracture (FNF) in old patients compared to internal fixation and hemiarthroplasty [9,10].

There are comparative studies on short versus standard stems in young patients with arthrosis [7], but there is a lack of information comparing clinical and radiological outcomes between standard and shorter stems in elderly patients with FNF. We prospectively assessed clinical outcomes—including walking performance, thigh pain, subjective evaluation, and radiographic findings—with particular attention to signs of stem stability and osseointegration. We conducted a prospective comparison of a short femoral stem (SFS) with a conventional standard stem (CSS) in patients with femoral neck fractures, with a minimum follow-up of two years.

## 2. Materials and Methods

### 2.1. Study Design

For this single-center prospective case–control study, Institutional Review Board approval was obtained (HJMPP 06/2020), and informed consent was obtained from all participants. Patients treated prospectively with SFS due to proximal femoral fracture were matched by sex and age, similar comorbidities and follow-up with an historic CSS group, with 1:2 proportion.

**Figure 1:**
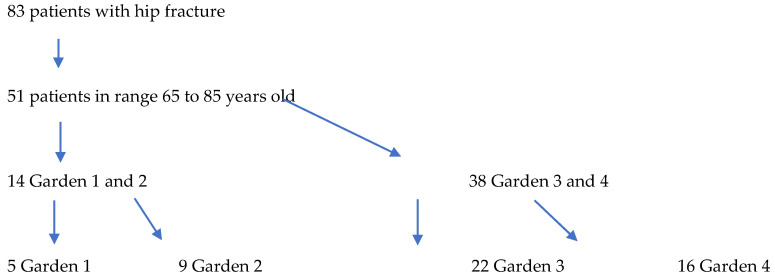
Chart flow.

Inclusion Criteria: Age greater than 65 years, an FNF Garden classification of 3–4, primary cementless THA, and follow-up for a minimum of 2 years. Exclusion Criteria: Previous hip surgery, neurologic impairment, older than 85 years, metabolic bone disease, and infection (Figure 1).

Demographic information, operative time, incision length, blood loss, duration of hospitalization, and any complications were documented. Clinical and radiological follow-up assessments were performed at 45 days, 3 months, and 6 months and subsequently on an annual basis.

### 2.2. Surgical Technique

**Figure 2:**
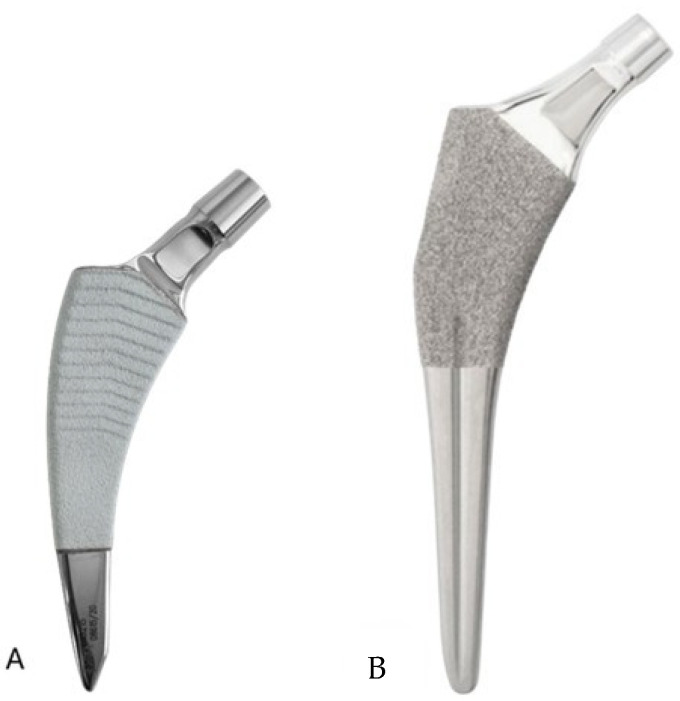
Photos of the short and conventional stems. (**A**) The proximal 2/3 of the Exacta RS short stem is double-taper trapezoidal shape double-coated with plasma-sprayed titanium and hydroxyapatite. (**B**) The conventional Alteon Taper Wedge stem features an interconnected macropore titanium plasma spray coating on the proximal body.

All procedures were performed by joint replacement specialists using a standardized posterolateral approach with piriformis retention [11]. Epidural anesthesia was used in all but two cases. The femoral neck was recut 1.5 cm proximally to the lesser trochanter. Acetabular preparation was performed by reaming until reaching the templated external diameter and exposing the subchondral bone. The definitive acetabular component—a highly porous, uncemented cup (JUMP^®^ System TRASER^®^, Permedica S.p.A., Merate, Italy)—was implanted following recommended orientation guidelines [12], using a polyethylene liner with a 10° posterior lip. Two screws were routinely placed for additional fixation. Femoral preparation was carried out using progressively sized compaction reamers. The short femoral stem (SFS) study group received Exacta RS^®^ (Permedica S.p.A.), introduced on the market in 2021. This collarless calcar loading stem is characterized by a triple-taper trapezoidal shape (Figure 2A). Its angulated distal end is in contact with the lateral cortex, thus enhancing load transfer laterally, preventing varus tilting, and providing three-point fixation [7]. The conventional standard stem (CSS) control group received the Alteon^®^ Taper Wedge Femoral Stem (Exactech, Gainesville, FL, USA), designed to achieve immediate axial and rotational stability (Figure 2B). This single taper stem is characterized by a lateral flare design to evenly distribute loads in the proximal femur.

### 2.3. Postoperative Care

Antibiotic prophylaxis (1 g of cefazolin every 8 h × 3 doses) and thromboprophylaxis (40 mg of enoxaparin daily × 30 days) were administered and switched to regular antithrombotic medication if needed (i.e., atrial fibrillation). Mobilization was allowed on the first postoperative day with full weight bearing as tolerated.

Clinical evaluation included the Harris Hip Score (HHS), maximum score is 100 points, and it is interpreted as follows: less than 70 points is a poor result; 70–79 is average; 80–89 is good; and 90–100 is excellent [13]. The Roles and Maudsley satisfaction score was included too and it is characterized by a 4-point pain and limitation assessment, where 1 is excellent with no pain or limitations after treatment, 2 is with significant improvement after treatment, 3 is relative improvement after treatment, and 4 is poor with similar or worse symptoms after treatment [14].

Radiographic evaluation included assessment of cup anteversion and inclination, using the Ackland method. Acceptable anteversion was between 0° and 15°. Acetabular inclination was measured using the angle between a line passing through the 2 vertices of the distal endpoint of the teardrop sign and the axis of the acetabular component [15]. Moreover, osseointegration was evaluated for both cup and stem, based on Moore’s criteria [16]. Radiolucent lines around the acetabular component were classified according to the DeLee–Charnley zones [17] and those around the femoral stem according to the Gruen zones [18]. Cup loosening was defined radiographically as a change in cup tilt greater than 5°, migration exceeding 2 mm, the presence of a radiolucent line thicker than 1 mm across all three zones on sequential radiographs, and/or evidence of screw breakage. Stem subsidence was measured from the shoulder of the stem to the lesser trochanter [19]. Leg length discrepancy (LLD) was determined using weight-bearing anteroposterior (AP) pelvic radiographs by drawing a line along the inferior borders of the teardrops and measuring the vertical distance from this line to the most prominent point of each lesser trochanter (teardrop method) [20]. Heterotopic ossification was classified according to the Brooker system [21].

### 2.4. Statistical Analysis

Data were analyzed using SPSS v20 (IBM Corp., Armonk, NY, USA). Continuous variables were compared using Student’s *t*-test and categorical data with the Chi-square test. A *p*-value less than 0.05 was considered statistically significant.

## 3. Results

Thirty-eight patients who received an SFS for femoral neck fracture were matched to a control group at a 1:2 ratio. In total, 114 patients (114 hips) were included in the analysis—38 in the SFS group and 76 in the CSS group—with a mean follow-up of 4.1 years (range, 2–5). The mean age was 73.1 years (range, 65–83) in the study group and 72.9 years (range, 65–82) in the control group.

Most patients in both groups were classified as ASA grade II on preoperative assessment, with no significant difference between groups (*p* = 0.752). The mean body mass index (kg/m^2^) was 26.7 ± 3.7 in the SFS group and 27.0 ± 3.1 in the CSS group, also without a statistically significant difference (*p* = 0.209) (Table 1).

One patient in the CSS group died after 3.5 years as a result of causes not related to the implant. Radiographic and clinical results were good at the last follow-up.

### 3.1. Intraoperative Outcomes

The mean operative time was 49.5 ± 4.7 min in the SFS group, with skin incisions ranging from 11 to 16 cm (average, 14.5 cm). In the CSS group, the mean operative time was 50.2 ± 4.3 min, and incision length ranged from 12 to 17 cm (average, 15.0 cm). Mean intraoperative blood loss was 385 ± 70 mL in the SFS group and 400 ± 50 mL in the CSS group; a total of nine patients required postoperative blood transfusion (three in the SFS group and six in the CSS group). The average length of hospital stay was 5.6 ± 1.5 days (range, 4–8) for the SFS group and 5.7 ± 1.0 days (range, 4–8) for the CSS group. No neurovascular complications were observed after the surgery, and one patient in the CSS group suffered deep vein thrombosis. There were three intraoperative fractures: One patient in the SFS group and one in the CSS group had a fracture in the calcar, which was fixed with cerclage wires. A third patient in the control group had a lesser trochanter crack, fixed with cerclage wires, and a disruption on the greater trochanter that was only observed on postoperative X-ray. All intraoperative fractures were monitored during the follow-up, showing no progression and not leading to stem instability. There were no statistically significant differences between the groups regarding intraoperative parameters (Table 2).

### 3.2. Clinical Outcomes

At the final follow-up, the mean postoperative HHS was 87 ± 2.7 points (range, 86–94) in the SFS group and 88 ± 2.5 points (range, 86–95) in the CSS group (*p* = 0.231). Regarding the Roles and Maudsley patient satisfaction score, all but one patient in the SFS group reported an excellent outcome, whereas in the CSS group, 64 patients rated their result as excellent and 12 as good. Thigh pain was evaluated during follow-up. At 3 months, three SFS and five CSS patients had symptoms during daily activities. At 6 months, one patient in SFS group had pain only during long-distance walking without a cane, and one in the CSS group had pain when climbing stairs. At the final follow-up, no patient reported pain in SFS group, while one patient in the CSS group did (during initial standing up and walking 10 steps (Table 3).

There were no significant differences in ambulatory function between the two groups preoperatively or at the 1- and 2-year follow-ups. However, in both groups, walking ability at follow-up had significantly declined compared with preoperative levels. Even though all patients returned to outdoor activities at the last follow-up, walking assistance with a cane was required by 11 with an SFS (due to fear of falling (7) and 4 other joint pathology (4)) and in 21 with a CSS (due to fear of falling (11), other joint pathology (9), and stroke (1)).

During the follow-up, there were no implant-related complications nor failures that required revision. One patient in the CSS group required a DAIR 21 days after surgery due to wound hematoma and recovered with no other complications. One patient in the SFS had a dislocation reduced under anesthesia.

### 3.3. Radiographic Outcomes

**Figure 3:**
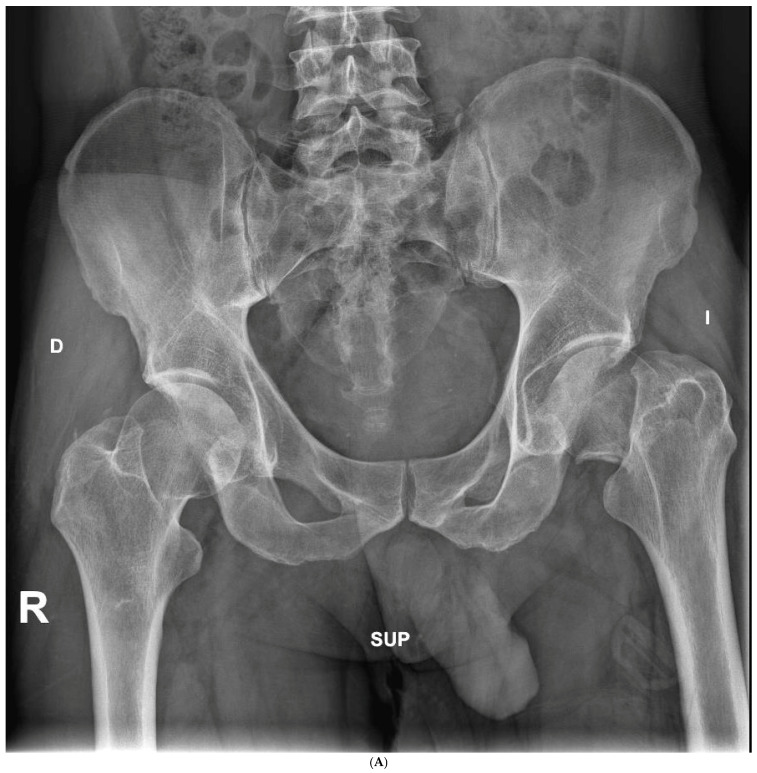

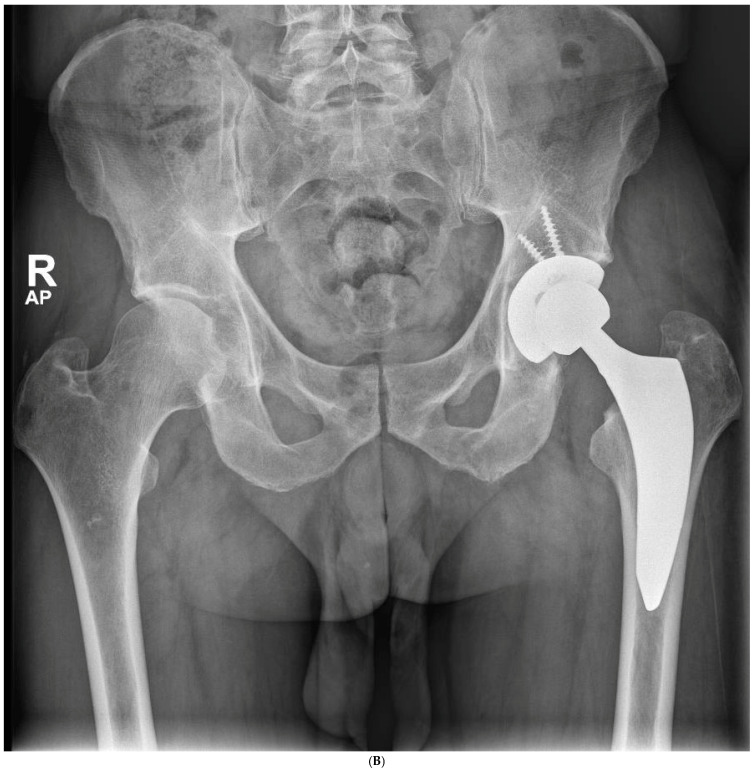

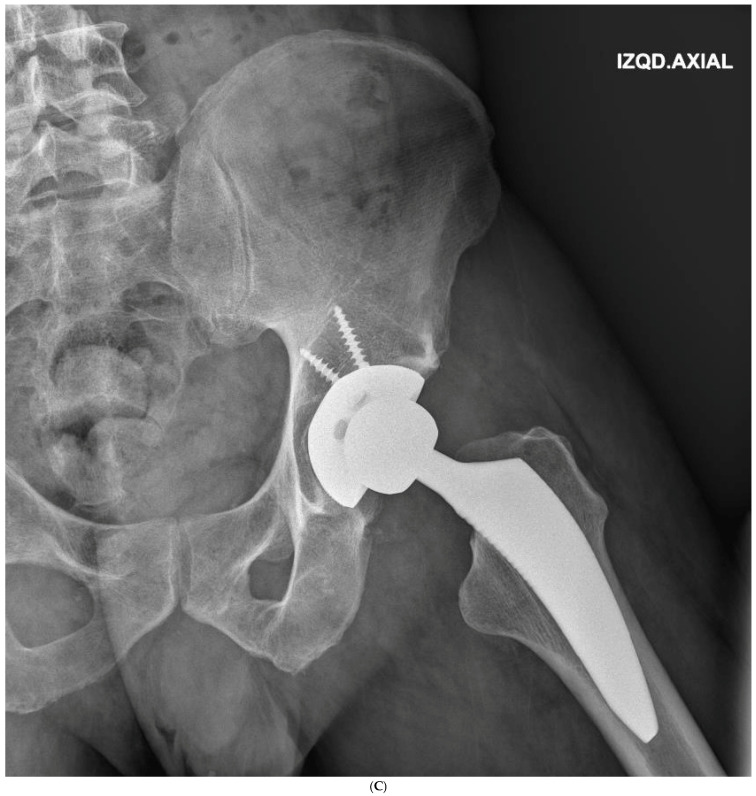
(**A**) Preoperative anteroposterior X-ray. Left hip sustained a displaced femoral neck fracture. (**B**) Postoperative anteroposterior X-ray (SFS group). Both the short stem and acetabular cup appear well osseointegrated and stable at final follow-up. (**C**) Postoperative lateral X-ray (SFS group). The stem is osseointegrated and there is absence of radiolucent lines at bone-implant interface.

**Figure 4:**
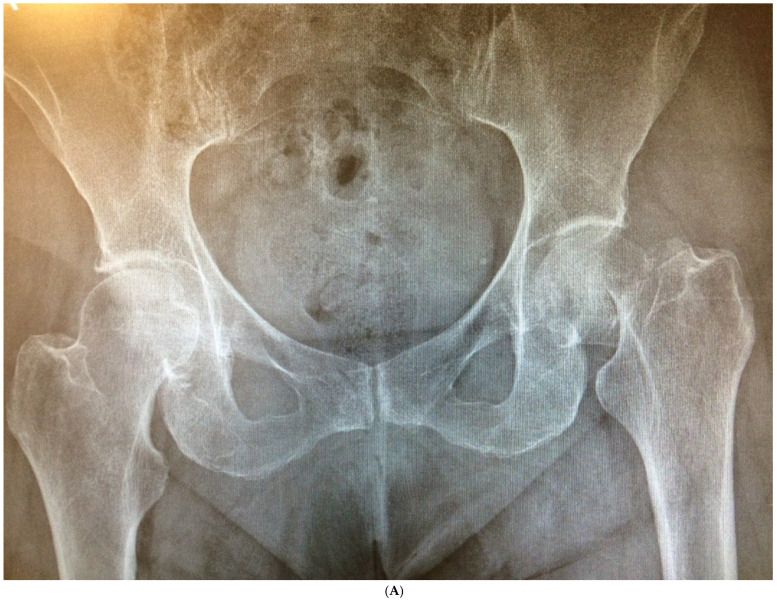

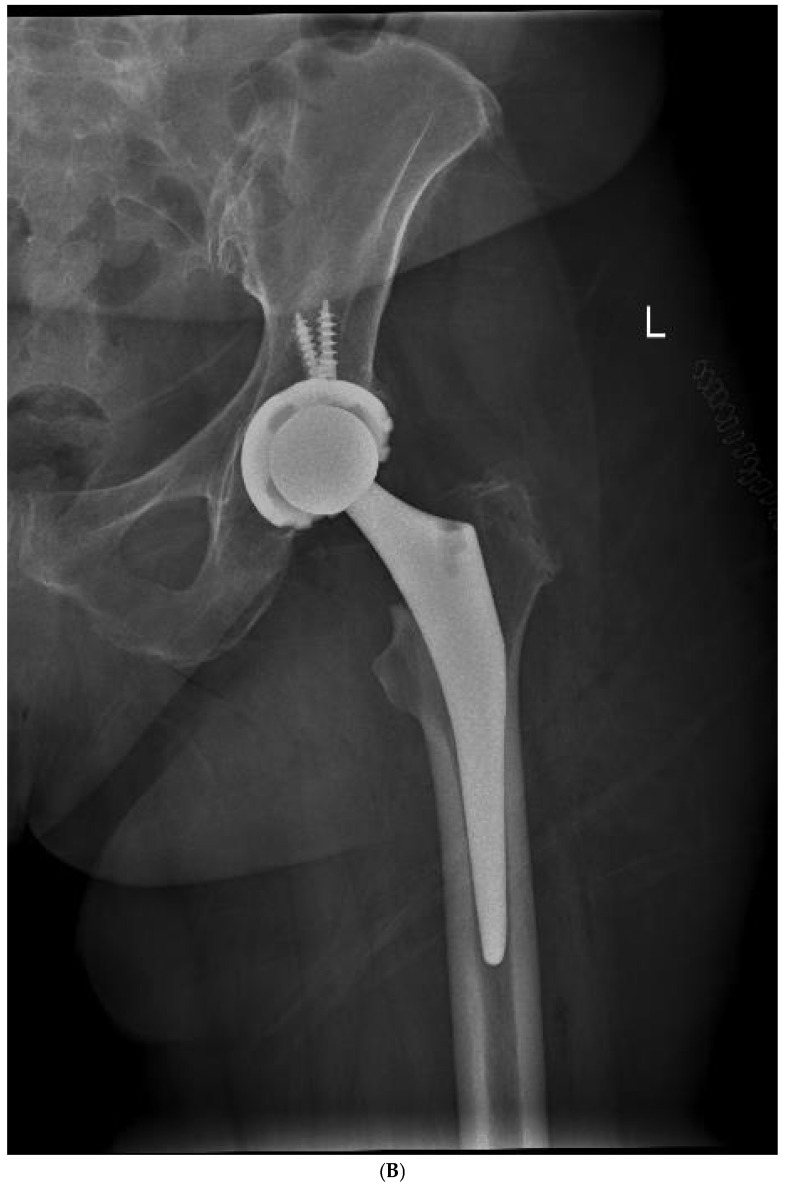

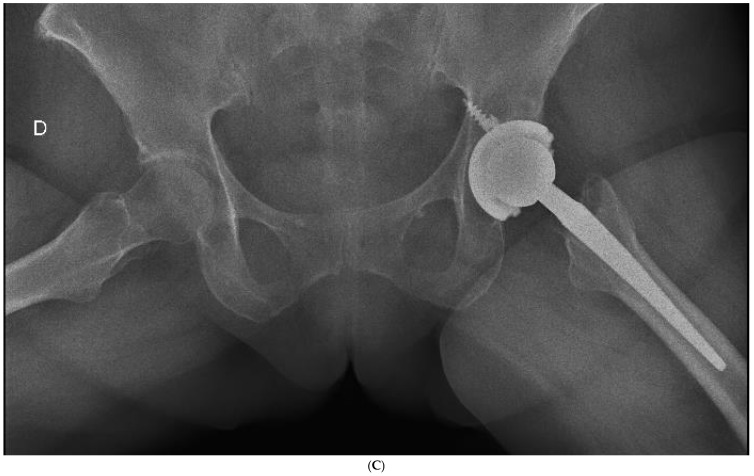
(**A**) Preoperative anteroposterior X-ray. Left hip sustained a displaced femoral neck fracture. (**B**) Postoperative anteroposterior X-ray (CSS group). Femoral stem shows osseointegration and good proximal loading, with no bone resorption at final follow-up. (**C**) Postoperative lateral X-ray (CSS group). At final follow-up, the stem shows no subsidence and good fixation.

The average cup inclination and anteversion were 43° ± 3° (range, 38–50°) and 10° (range, 0–15°), respectively, in the SFS group, compared with 44° ± 5° (range, 37–50°) and 5° (range, 3–15°) in the CSS group. All acetabular components were positioned within Lewinnek’s safe zone. No radiolucent lines, cup migration, or broken screws were observed in either group throughout the follow-up period. Radiographic signs of osseointegration were present in all implants, with at least three of Moore’s criteria met in every SFS case (Figure 3A–C), indicating complete osseointegration. Similar findings were noted in the CSS group (Figure 4A–C). No femoral radiolucency or periprosthetic osteolysis was detected at any scheduled follow-up. Stem subsidence of approximately 3 mm was observed at the 3-month follow-up in two hips in the SFS group and three hips in the CSS group; however, these cases were not associated with clinical symptoms or functional impairment. No femoral stem loosening was identified during the follow-up period. A leg length discrepancy (LLD) of less than 1 cm was observed in three hips in the SFS group (average, 4 ± 3 mm) and in eight hips in the CSS group (average, 4 ± 5 mm). None of these discrepancies resulted in clinical or functional complaints, and no heterotopic ossification was identified in the radiographic evaluations (Table 4).

## 4. Discussion

This study shows that in elderly patients undergoing THA for displaced femoral neck fracture a modern metaphyseal-anchoring short stems perform equivalently to standard-length cementless stems with respect to function, patient satisfaction, radiographic fixation, and complications over a minimum of two years.

Clinical outcomes were similar between the two groups, with no significant differences found in the HHS or the Roles and Maudsley score. Our results are consistent with previous studies reporting comparable clinical outcomes and PROMs after THA using short femoral stems [4,22]. Additionally, the use of a posterior surgical approach did not influence these outcomes [23].

Our findings are consistent with the recent literature demonstrating excellent survivorship and stable osseointegration in patients with an SFS, even in patients with compromised bone quality. Kutzner et al. prospectively evaluated calcar-guided short stems in elderly FNF patients and reported a stem survival of 96.2% with excellent osseointegration [24]. Similarly, Zimmerer et al. compared short and conventional stems and found no significant differences in functional outcomes or radiographic stability over mid-term follow-up [25]. Lee SJ et al. reported complete osseointegration in their series of 38 patients with fractures and osteoporotic bone treated with a short stem [4].

The low rate of thigh pain in our short-stem cohort supports biomechanical data suggesting improved metaphyseal load transfer and reduced distal stress shielding. Recent finite-element studies confirm that short stems redistribute forces more physiologically in osteoporotic femora, reducing micromotion and strain concentration [4,26].

Radiographically, our stems demonstrated stable osseointegration with no loosening or osteolysis—in parallel with the findings of Mauch et al., who documented secure fixation of short stems in both primary and revision settings [27]. Subsidence of up to 3 mm, observed occasionally in our series, is regarded as a benign early adaptive settling and is widely reported across modern cementless stems [28]. Additionally, LLD and anatomical reconstruction were excellent in our study, with no significant discomfort and no clinical relevance in all patients [29].

Several studies have advised against using cementless fixation in elderly patients because of the higher risk of periprosthetic femoral fractures (PFFs) [30]. However, recent evidence (including the findings of our study) indicates that cementless short femoral stems (SFSs) are associated with a low incidence of periprosthetic femoral fractures and may even present a reduced risk compared with conventional cementless straight stems [5,31]. Notably, Gkagkalis et al. [22] reported no overall increase in fracture rates with the Optimys^®^ short stem (Mathys, Bettlach, Switzerland), observing rates of 1.5% in patients younger than 60 years and 1.4% in patients older than 75 years at the time of surgery.

A recent study from Steinbrück et al. [32] demonstrated similar 5-year overall revision rates between matched cohorts of short-stem THAs (2.9% CI 2.4–3.5) and standard-stem THAs (3.1%, CI 2.7–3.4), using data from the German Arthroplasty Registry and similar to those from the Australian National Joint Replacement Register [33]. At final follow-up, our results showed a better performance with no revisions. Data from the national registers are now arising about the option to use uncemented collared stems for hip fractures and osteoporotic bone, have at similar follow up good performance and lowering complications as periprosthetic fractures [32,33].

The importance of our study is the information about a specific implant, in a different view of registers where no prosthesis-specific results were described. The Australian National Joint Replacement Register reported initial similar evaluation at 1 year and a lower revision incidence for SFS at 13 years [33].

This study has several limitations. First, although the sample size is relatively small, the comparison of patients with the same pathology and similar age enhances the validity of the conclusions drawn from the results. Second, the mean follow-up of 4 years limits the assessment of long-term implant survival and revision rates; however, the radiographic findings are encouraging, as all implants demonstrated clear signs of osseointegration according to Moore’s criteria, along with excellent clinical outcomes. Third, stem subsidence was assessed using anteroposterior radiographs rather than a more precise method such as radiostereometric analysis.

## 5. Conclusions

Short femoral stems in total hip arthroplasty for femoral neck fractures provide excellent clinical and radiographic outcomes comparable to those of conventional-length stems, with excellent early implant survival, a low rate of complications and revisions, and no radiographic signs of aseptic loosening. Their bone-preserving design and reliable fixation make short stems a valuable option in patients with long life expectancy and a high risk of revision during their lifetime.

## Figures and Tables

**Table 1:** Demographics and baseline characteristics. (HTA, arterial hypertension; DMTS, disease-modifying therapies; n.s., not significant).

Variable	SFS Group (*n* = 38)	CSS Group (*n* = 76)	*p*-Value
Age, years (mean ± SD, range)	73.1 (65–83)	72.9 (65–82)	n.s.
Sex distribution	22 female/16 male	37 female/29 male	0.582
BMI (kg/m^2^)	26.7 ± 3.7	27.0 ± 3.1	0.209
ASA grade II (%)	24 (63%)	54 (59%)	0.752
Comorbidities	HTA 11 (29%) DMTS 7 (18%) Cardiac disease 3 (8%)	HTA 21 (28%) DMTS 11 (15%) Cardiac disease 8 (10.5%)	n.s.

**Table 2:** Intraoperative and early postoperative outcomes. (DVT, deep vein thrombosis; DAIR: debridement, antibiotics, implant retention; n.s., not significant).

Variable	SFS Group (*n* = 38)	CSS Group (*n* = 76)	*p*-Value
Operative time (min)	49.5 ± 4.7	50.2 ± 4.3	n.s.
Incision length (cm)	11–16 (mean 14.5)	12–17 (mean 15)	n.s.
Intraoperative blood loss (mL)	385 ± 70	400 ± 50	n.s.
Postoperative transfusions (n)	3	6	n.s.
Hospital stay (days)	5.6 ± 1.5 (4–8)	5.7 ± 1 (4–8)	n.s.
Neurovascular injury	0	0	n.s.
Intraoperative fractures	1 (lesser trochanter)	2 (lesser + 1 greater trochanter)	n.s.
Early postoperative complications	1 dislocation	1 DVT; 1 DAIR for hematoma	n.s.
Mortality during follow-up	0	1 (at 3.5 years)	n.s.

**Table 3:** Comparison of clinical outcomes: SFS vs. CSS group. (n.s., not significant).

Clinical Measure	SFS Group (*n* = 38)	CSS Group (*n* = 76)	*p*-Value
Final HHS (mean ± SD)	87 ± 2.7 (range: 86–94)	88 ± 2.5 (range: 86–95)	0.231
Roles and Maudsley satisfaction	37 excellent, 1 good	64 excellent, 12 good	n.s.
Thigh pain at 3 months	3 cases	5 cases	n.s.
Thigh pain at 6 months	1 (mild)	1 (stairs)	n.s.
Thigh pain at final follow-up	0	1 (early standing)	n.s.
Cane use at final follow-up	11 patients (7 due to fear of falling, 4 due to joint pathology)	21 patients (11 due to fear of falling, 9 due to joint pathology, 1 due to stroke)	n.s.
Implant-related complications	0	0	n.s.
Dislocation	1 (successfully reduced)	0	n.s.

**Table 4:** Radiographic Outcomes and Statistical Comparison Between SFS and CSS Groups (n.s., not significant; n.a., not applicable due to absence of events in both groups).

Parameter	SFS Group	CSS Group	*p*-Value
Cup inclination mean ± SD (range)	43° ± 3° (38–50°)	44° ± 5° (37–50°)	n.s.
Cup anteversion mean (range)	10° (0–15°)	5° (3–15°)	n.s.
Cup position within Lewinnek’s safe zone	100%	100%	n.a.
Radiolucent lines/cup migration/broken screws	None observed	None observed	n.a.
Radiographic osseointegration	100%; ≥3 Moore’s criteria in all cases	100%	n.a.
Femoral radiolucency or periprosthetic osteolysis	None detected	None detected	n.a.
Stem subsidence (~3 mm at 3 months)	2 hips, asymptomatic	3 hips, asymptomatic	n.s.
Femoral stem loosening	None observed	None observed	n.a.
Leg length discrepancy (LLD)	3 hips; mean 4 ± 3 mm (<1 cm)	8 hips; mean 4 ± 5 mm (<1 cm)	n.s.
Clinical impact of LLD	None	None	n.a.
Heterotopic ossification	None observed	None observed	n.a.

## Data Availability

The original contributions presented in this study are included in the article. Further inquiries can be directed to the corresponding author.
